# Roy’s Adaptation Model-Based Patient Education for Promoting the Adaptation of Hemodialysis Patients

**DOI:** 10.5812/ircmj.12024

**Published:** 2013-07-05

**Authors:** Ardashir Afrasiabifar, Zohreh Karimi, Parkhideh Hassani

**Affiliations:** 1 Department of Nursing, Yasuj University of Medical Sciences, Yasuj, IR Iran; 2 Department of Nursing, Ahvaz Jundishapur University of Medical Sciences, Ahvaz, IR Iran; 3 Department of Nursing, Shahid Beheshti University of Medical Sciences, Tehran, IR Iran

**Keywords:** Kidney Failure, Chronic, Renal Dialysis, Adaptation, Roy’s Adaptation Model

## Abstract

**Background:**

In addition to physical adaptation and psychosocial adjustment to chronic renal disease, hemodialysis (HD) patients must also adapt to dialysis therapy plan.

**Objectives:**

The aim of the present study was to examine the effect of Roy’s adaptation model-based patient education on adaptation of HD patients.

**Patients and Methods:**

This study is a semi-experimental research that was conducted with the participation of all patients with end-stage renal disease referred to the dialysis unit of Shahid Beheshti Hospital of Yasuj city, 2010. A total of 59 HD patients were randomly allocated to two groups of test and control. Data were collected by a questionnaire based on the Roy’s Adaptation Model (RAM). Validity and reliability of the questionnaire were approved. Patient education was determined by eight one-hour sessions over eight weeks. At the end of the education plan, the patients were given an educational booklet containing the main points of self-care for HD patients. The effectiveness of education plan was assessed two months after plan completion and data were compared with the pre-education scores. All analyses were conducted using the SPSS software (version 16) through descriptive and inferential statistics including correlation, t-test, ANOVA and ANCOVA tests.

**Results:**

The results showed significant differences in the mean scores of physiological and self-concept models between the test and control groups (P = 0.01 and P = 0.03 respectively). Also a statistical difference (P = 0.04) was observed in the mean scores of the role function mode of both groups. There was no significant difference in the mean scores of interdependence modes between the two groups.

**Conclusions:**

RAM based patient education could improve the patients’ adaptation in physiologic and self-concept modes. In addition to suggesting further research in this area, nurses are recommended to pay more attention in applying RAM in dialysis centers.

## 1. Background

Glomerular Filtration Rate (GFR) is reduced by up to 10% of its normal level in end stage renal disease (ESRD). Uremia occurs due to kidney inability to eliminate toxin waste products. Uremic syndrome may result in patient’s death if he/she does not undergo dialysis therapy or is unable to perform kidney transplantation. Since all patients with ESRD are not able to undergo kidney transplantation, many of them are subject to long-term dialysis therapy. Blood dialysis or hemodialysis (HD) is the most common type of dialysis therapy worldwide (1). Although Iran has the first rank of kidney transplantation in the Middle East, yet, there are great problems in accessing donor kidney transplants. There are about 15000 ESRD patients in Iran, the majority of which are HD patients (2-4). HD patients experience numerous physical and psychosocial stressors (5, 6) including fatigue, limited physical activities, dietary and fluid restriction, chronic pains, muscular cramp, depression and anxiety, uncertain future, and conflicts regarding life and death (7-13). Low quality of life of HD patients (14, 15) could be associated with increased mortality, cardiovascular incidence and hospitalization (16). In spite of the useful effects of dialysis therapy, it can itself act as a source of stress. HD patients call themselves dialysis patients who have to adapt with the dialysis program. Adherence to a dialysis plan, invasive procedures, duration of dialysis therapy, prescribed medicines, age, sex, level of education, job, social support, and coping mechanisms (17-20) are factors which affect the patient’s adaptation. Nurses’ knowledge of these factors and adaptation models will enable them to help the patients to adapt effectively to the illness.

So far, different models for adaptation to chronic illness have been suggested. Bio-medical models mainly pay attention to physical adaptation and physiological consequences, while psychological ones put emphasis on behavioral reactions and psychic outcomes. In spite of the positive benefits of these models, they have particular limitations for clinical application (21). In parallel to bio-psychosocial models; nursing models have also been introduced by nursing scholars who emphasize on the holistic concept of the human being. Among nursing models, Roy’s Adaptation Model (RAM) is one, which would form the conceptual framework of the present study. Application of this model has been interesting for nursing researchers in clinical settings (22-25). Adaptation is the main concept of RAM and the goal of nursing is promotion of adaptation in each of the four modes namely physiological, self-concept, role function and interdependence. Nursing interventions contribute to restoration and maintenance of the patient’s adaptation through changing or manipulating the internal and external stimuli (26). The nursing care program of ESRD is a complex plan. It not only includes dialysis therapy but also involves fundamental changes in life style for facilitating the patient’s adaptation to chronic renal disease and its consequences. Ineffective adaptation reduces quality of life and increases treatment expenses. Numerous studies have investigated the effects of patient education on physical or psychological outcomes in ESRD, but only a few researches investigating the effects of RAM-based interventions in HD patients have been conducted. The patients’ frequent attendances to dialysis wards make an opportunity to implement nursing model-based education. This would increase patient knowledge about self-care behaviors, coping with renal disease and adaptation to the long-term dialysis plan.

## 2. Objectives

The aim of the present study was to examine the effect of RAM-based education on the adaptation of HD patients.

## 3. Patients and Methods

This semi-experimental study was conducted with the aim of improving the adaptation of HD patients using RAM-based patient education. The research population included all HD patients referred to the dialysis ward of Shahid Beheshti Hospital of Yasuj, Iran during 2010. This hemodialysis center had 64 HD patients but 59 patients had the eligibility criteria of the study and 5 patients were excluded. Eligible HD patients were selected using the convenience sampling method but randomly divided into two groups of test and control. Inclusion criteria were final diagnosis of ESRD, being at least three months under HD, aged 18 to 75 years, no kidney transplant case, no known case of psychiatric disorders or handicaps, having at least writing and reading literacy and lack of previous participation in studies like this research. Unwilling to participate in the study, undergoing kidney transplantation and migrating from the place of research were considered as the exclusion criteria. Sampling was initiated after explaining the purpose of the study, taking informed consent and receiving permission from relevant authorities. The confidentiality of the collected data was emphasized and participants were reminded that they could withdraw from the study at any time. The study was approved by the Research Ethics Committee of Yasuj University of Medical Sciences and registered with the local code as ECC23217 on 24^th^ of May 2010. According to RAM, patient assessment was conducted at two levels: behaviors and stimuli assessment for four physiological, self-concept, role-function and interdependence modes. Nursing diagnosis and the goal setting was identified based on ineffective behaviors and its related stimuli. Nursing education was then implemented. It includes education before and during dialysis and if a patient needed expert consultation, he/she was referred to a specialist. The education plan contained information about kidney function and its role in body homeostasis, etiology, diagnosis, treatment and complications of kidney failure, self-care regarding nutrition, sleep, rest, activity, fluid and electrolytes balance, elimination, the arterovenous fistula, skin/nail health, positive thinking, and interaction with family, friends and significant others. The plan was held for eight one-hour sessions during eight weeks. At the end of the plan, they were given an educational booklet containing the main points of self-care for HD patients. The effectiveness of the plan was evaluated two months after completion of the program and the result was compared with the pre-education data.

The data were collected by a questionnaire containing items regarding the demographic variables and four adaptation modes. The physiological mode was assessed by 25 questions, using a five-item scale (never, rarely, sometime, often and always) related to oxygenation, nutrition, elimination, activity and rest, senses, protection, fluid and electrolyte balance, and neuroendocrine functions. Minimum and maximum scores of physiological adaptation were 25 and 125, respectively. For the self-concept mode, 10 questions with a five-item scale (always, frequently, sometimes, rarely and never) were used to assess the patient’s ideas, thoughts and feelings about body image, body sensations, physical self, as well as personal self (self-consistency, self-ideal, self-ethic and self-spiritual). For this mode, the scores ranged between 10-50. Eight questions with a five-item scale (never, shortened period, sometimes, often and always) were used to assess the role function mode. The scores of this mode ranged between 8-40. Data related to the interdependence mode was collected through 6 questions with a five-item scale (fully disagree, disagree, no opinion, agree, and fully agree) about giving and receiving respect, love and value in their communications. The scores of this mode ranged between 6-30. The content of the questionnaire was set based on review of the available research articles, concepts of RAM and opinions of ten faculty members familiar with RAM. Item-level content validity index (I-CVI) was used to measure content validity. For each item ‘to be relevant”, “to be clear” and “to be simple” was assessed by five faculty members and questions with scores less than .75 were discarded. Test-retest reliability was used to confirm reliability of the questionnaire. The questionnaire was given to 10 HD patients to fill. It was again given to them two weeks later. The reliability for twice measurements, Cronbach’s Alpha coefficient was equal 0.80. All statistical analyses were performed using the SPSS statistical package, version 16. Descriptive statistics including central and dispersion statistics as well as a series of inferential statistics such as correlation, t-test, ANOVA and ANCOVA were used. A P < 0.05 was defined as statistically significant. The distribution of dependent variables was checked by Kolmogorov-Smirnov Z test. Parametric tests were used to compare the mean of outcome variables since their distributions were normal.

## 4. Results

Thirty-one patients in the test group and 28 subjects in the control group with the mean age of 47.35 ± 13.76 years (range, 21-70 years) participated in the present study; 52.5% were male and 47.5% were female. The average length of the disease from the time of diagnosis of ESRD was 33.93 ± 29.23 months. The majority of patients had undergone HD 3-4 times per week and the mean duration of HD sessions was 3.7 ± 1.3 hours. 68.8% said that they did not know much about chronic renal failure and 85.5% mentioned that dialysis nurses were the most important source of their information. Table 1 shows the demographic characteristics of both groups and independent samples t test, Chi-square and Fisher’s exact test show no significant statistical differences between the two groups. 

**Table 1.** Demographical Data of the Test and Control Groups

Group Variable	Test 31 (52.5%)	Control 28 (47.5%)	P value
**Age, Mean ± SD**	48.03 ± 13.79	46.86 ± 14.36	P = 0.63 ^a^
**Duration of HD sessions, Mean ± SD**	3.52 ± 1.79	3.48 ± 1.72	P = 0.33 ^a^
**Gender, No (%)**			P = 0.41 ^b^
Male	16 (51.6%)	15 (53.6%)	
Female	15 (48.4%)	13 (46.4%)	
**Marital Status, No (%)**			P = 0.50 ^b^
Single	26 (88.9%)	22 (78.6%)	
Married	5 (11.1%)	6 (21.4%)	
**Education Level, No (%)**			P = 0.45 ^b^
Diploma >	25 (80.6%)	21 (75%)	
Diploma ≤	6 (19.4%)	7 (25%)	
**Place of Residence, No (%)**			P = 0.40 ^b^
City	13 (41.9%)	12 (42.8%)	
Village	18 (58.1%)	16 (57.2%)	
**HD Sessions per Week, No (%)**			P = 0.31 ^b^
2 ≤	11 (34.5%)	12 (42.8%)	
3-4	30 (65.5%)	16 (57.2%)	
**Length of Starting HD, No (%)**			P = 0.28 ^c^
1 year <	8 (25.9%)	10 (35.7%)	
1-2 years	10 (32.2%)	10 (35.7%)	
> 2 years	13 (41.9%)	8 (28.6%)	
**Employment No (%)**			P = 0.27 ^b^
Yes	6 (19.3%)	7 (25%)	
No	25 (80.7%)	21 (75%)	

^a^independent t test

^b^ Fisher’s Exact test

^c^P value based on Chi-square

According to the results of the independent samples t test, no significant differences were found for the mean scores of adaptation levels in the test and control groups before the education. But t-independent test revealed a statistical difference for the mean scores of physiologic (P = 0.001), self-concept (P = 0.03) and role-function modes (P = 0.04) between the two groups after the education. Also t-paired test showed a significant difference in the mean scores of physiologic, self-concept and role-function modes for the test group before and after education (Table 2). 

**Table 2.** Comparison of the Mean Scores of Adaptation Modes of Both Groups

Group Adaptation Modes, Mean ± SD	Test				Control			
	Pre-intervention	Post-intervention	P value	Paired Diff of Mean	Pre-intervention	Post-intervention	P value ^b^	Paired Diff of Mean
**Physiologic**	68.76 ± 13.8	79.15 ± 3.70	0.01 ^a^	-10.39	68.65 ± 13.41	69.55 ± 13.9	0.06	-0.9
**Role-function**	29.75 ± 5.39	32.93 ± 5.48	0.03 ^a^	-3.18	29.40 ± 5.45	29.96 ± 5.50	0.1	-0.29
**Self-concept**	33.31 ± 9.96	38.96 ± 10.07	0.01 ^a^	-5.65	33.87 ± 9.46	34.03 ± 9.31	0.35	-0.16
**Interdependence**	23.2 ± 5.68	24.68 ± 5.71	0.06	-1.48	23.03 ± 5.61	23.78 ± 5.63	0.07	-0.75

^b^ t test: significant at the P < 0.05

^a^ P value based on paired samples

There was no significant correlation between age and the scores of adaptation modes. Independent samples t test showed a significant difference for the mean scores of self-concept between the male and female patients of both groups on pre and post education (Table 3). 

**Table 3.** The Mean Scores of the Four Adaptation Modes of the Two Groups in Terms of Gender

Group	Gender		P value ^b^
	Male	Female	
**Control**			
**Physiologic**			
Pre-intervention	69.10 ± 14.83	68 ± 11.57	P = 0.82
Post-intervention	70.36 ± 15.78	69.84 ± 11.24	P = 0.76
**Role function**			
Pre-intervention	28.78 ± 6.39	30.30 ± 3.77	P = 0.44
Post-intervention	29.21 ± 6.23	31.07 ± 4.21	P = 0.35
Self-concept			
**Pre-intervention**	32.47 ± 10.79	35.72 ± 6.99	P = 0.01 ^a^
Post-intervention	32.42 ± 10.51	36.38 ± 6.93	P = 0.04 ^a^
**Interdependence**			
Pre-intervention	21.00 ± 5.17	22.00 ± 5	P = 0.07
Post-intervention	21.84 ± 5.19	23.61 ± 5.22	P = 0.06
**Test**			
**Physiologic**			
Pre-intervention	67.41 ± 14.28	70.53 ± 13.28	P = 0.53
Post-intervention	76.58 ± 14.64	80.93 ± 13.09	P = 0.50
**Role function**			
Pre-intervention	28.88 ± 5.13	31.89 ± 5.22	P = 0.11
Post-intervention	35.05 ± 5.28	35.06 ± 5.34	P = 0.14
**Self concept**			
Pre-intervention	30.64 ± 11.24	36.13 ± 7.81	P = 0.03 ^a^
Post-intervention	36.17 ± 11.20	42.41 ± 14.78	P = 0.04 ^a^
**Interdependence**			
Pre-intervention	23.58 ± 4.89	24.63 ± 5.27	P = 0.2
Post-intervention	24.21 ± 4.80	25.53 ± 5.20	P = 0.2

^b^ t test: significant at the P < 0.05

^a^ P value based on independent samples

No significant correlation was observed between the scores of the four adaptation modes and variables such as level of education and numbers of HD sessions per week. Independent t test showed significant differences in the scores of physiologic and role function modes, pre-education in terms of variables such as marital status, employment status, residence (P < 0.05). One Way ANOVA showed statistical differences in the scores of physiologic and role function modes pre-education in terms of duration of HD and length of diagnosis of ESRD (P < 0.05). ANCOVA test showed significant difference between the two groups after eliminating the effects of duration of HD and length of diagnosis of ESRD as confounding variables.

## 5. Discussion

The results showed that RAM-based education plan had been able to improve the adaptation of some modes in HD patients. According to RAM, coping processes consist of two regulator and cognator subsystems. Proper response of these subsystems to external and internal stimuli results in adaptive behaviors (26). This finding could be consistent with studies investigating the effectiveness of educational plans in cardiac failure and chronic pulmonary disease (27, 28). Patient education can improve illness perception, support self-care behaviors and help acquire skills to deal with the chronic nature of renal disease. It must be, therefore, considered as an important part of ESRD treatment (29). The results showed that physiological adaptation was improved following nursing education. There are controversy findings in this regard. The review of available literature showed that HD educational plans were mainly focused on providing disease-related information and adhering to the medical regimen. Also most of them reported positive physiological effects following patient education plans. On the other hand, the effectiveness of traditional methods was criticized and shifts towards approaches including empowering and self-care management was suggested (30). Ford et al. examined the efficacy of dietary education on the laboratory parameters and knowledge of 63 HD patients at the completion of plan and six months later. Their study showed an increase in knowledge for both stages but the level of phosphorus had decreased only for the first stage of the study (31). The positive effect of exercise programs on physical function, cardio-respiratory capacity and activity tolerance time in HD patients was reported by other studies (32, 33). Also other studies showed an increased knowledge and reduced serum level of creatinine, uric acid and phosphates in Iranian ESRD patients following educational interventions (34, 35).

The present study showed that educational intervention had a significant on the patients’ self-concept. Similar findings regarding the impact of education on depression, self-efficacy, and physical and mental quality of life (36-38) confirm our results. This mode needs psychic integrity and is influenced by cognitive and affective factors (26). Personal beliefs system, family structure, socioeconomic status, type of personality, perceived social support and the patient’s perception of well-being (39) are the facilitators of or barriers of psychological adjustment to illness. In addition to the loss of a functional kidney, other losses such as losing family, vocational and social roles, physical skills, cognitive abilities (39), long process of treatment and obligations to extensive changes in life-style (40) could result in radical changes in the psychosocial welfare of HD patients.

Comparison of the mean scores of the role function mode showed a statistical difference, post-education, between the two groups, however, it seems that this difference was not clinically important. Unlike this finding, a significant improvement was reported in the role function of COPD patients after conducting RAM-guided patient education and the authors argued that patient education increases knowledge about the disease, better controls the symptoms and enhances well-being and consequently promotes the role function (27). Possible reasons for the lack of remarkable improvements in this mode may be due to unemployment of the majority of subjects (80%) and also HD patients’ employment needs further collaboration of multidisciplinary teams at national and local levels. Although role-function needs social integrity, yet available evidence shows that a high percentage of ESRD patients especially those who underwent long term dialysis therapy are unemployed or unable to return to their work (41, 42) and chronic renal disease may challenge their family roles and social responsibilities (43-45). Neuropathies, anemia, post dialysis fatigue, age (46, 47), the employment status before dialysis therapy, education level (48) and treatment modality (49) are influencing factors on the patient role function. In addition to financial benefits of employment, this matter is psychosocially important. Therefore, paying attention to the employment status, changing the sedentary life style, exercise training during the dialysis and administrating erythropoiesis-stimulating agents (43, 50) are essential for maintaining the social integrity of HD patients.

Nursing education could not promote the interdependence mode. This finding is consistent with the results of a similar study that reported no improvement in this mode post-education. This (27) may be due the majority of patients (81.35%) being single in this study. Also 57.63% of the patients live in rural regions in which the structures of family are nuclear and the parents live with children especially unmarried children. The interdependence mode needs for affection adequacy and includes giving and receiving love, respect and value in interpersonal interactions (26). HD patients also need emotional support in addition to informational and tangible or material support. Affective support could speed up access to health services, reduce anxiety and depression. High rate of emotional distress in ESRD patients (51) and a correlation between lack of affective support and patient survival rate have been reported (52). Suicidal behaviors were greater among single or divorced patients who had no emotional support from their family, friends and significant others (53). A study showed that HD women with high effective support form their husband had higher survival rates as well as better acceptance of the disease (52). Another study reported that relationships between family members are correlated with their affective support (18). HD patients may experience social isolation due to the chronic nature of their disease. Educational plans which would increase interpersonal interactions must be considered in their psychosocial management. Developing interpersonal communications such as peer groups could strengthen self-esteem and decrease dependency of HD patients to their family (54). HD patients should not be considered as a distinct therapeutic unit; rather they should be seen as the framework of an intimate couple, family member, friends, dialysis unit, neighbors and the community (39).

We selected a holistic approach containing biopsychosocial domains for educating HD patients but this study has several limitations. The sample size was small because we only had one dialysis ward in Yasuj city. In spite of allocating samples into two groups, it was not possible to randomly select the samples due to the small number of HD patients and this is not a characteristic of experimental studies. The validity and reliability of the questionnaire was approved but the lack of specific questionnaires on adaptation of HD patients is another limitation of the study. Therefore, generalization of the results needs to be more careful. The HD patients require basic changes in their lives for adaptation to ESRD and the dialysis plan. RAM-based patient education could improve the adaptation of HD patients in the physiological, self-concept and role function modes. The patients could be taught how to live with the disease and its limitations through nursing education. The results of this study show that the application of RAM for HD patients is possible due to their frequent presence in the dialysis units. The application of RAM can develop their adaptive abilities in the physical, psychosocial and spiritual domains. Economic problems resulting from ESRD may be inevitable, but physical and psychosocial problems may be modified through empowering the HD patients and the nurse’s roles are undeniable in this regard.
